# The labor market of digital labor platforms in Chile: companies, state regulation and workers

**DOI:** 10.3389/fsoc.2025.1673277

**Published:** 2025-09-30

**Authors:** Antonio Stecher, Karol Morales, Alan Valenzuela

**Affiliations:** ^1^Psychosocial Work Studies Program, Faculty of Psychology, Universidad Diego Portales, Santiago, Chile; ^2^Faculty of Psychology, Universidad Alberto Hurtado, Santiago, Chile

**Keywords:** digital labor platforms, labor market, Chile, platform workers, labor laws

## Abstract

**Introduction:**

In Chile, as in different Latin American countries, a growing expansion of digital labor platforms has been observed since 2010. The study of the labor markets of these platforms in countries such as Chile is of great importance to understand the particularities that these companies acquire in contexts other than the global north. Contexts with high rates of informal employment and fragile labor institutions give rise to particular dynamics and configurations in these labor markets. In turn, the case of Chile is of special interest due to the recent approval of a law (21.431)—the first of its kind in Latin America- regulating the contract of delivery and transportation platform workers, as well as its pioneering role in the processes of neoliberal modernization in the region, which are at the basis of many of the current productive and labor dynamics of the platforms.

**Methods:**

The reconstruction of the delivery and ride-hailing digital platforms labor market is based on the analysis of data (interviews with workers, interviews with key informants, documentary analysis of primary sources, review of secondary sources, field observations) collected between 2019 and 2025 in the framework of three research projects.

**Results and discussion:**

We present a reconstruction of the delivery and ride-hailing digital platforms labor market organized around three axes: (i) *platform companies*, (ii) *state labor regulations*, and (iii) *workers*. The analysis incorporates a diachronic perspective that highlights key milestones in the development of this labor market in Chile between 2010 and 2025, accounting for its dynamism and transformation. Likewise, a perspective is incorporated that accounts for the role of different actors—business actors, State actors (legislative, executive, and judicial branches) and worker organizations- in shaping the labor market of digital work platforms in Chile. The article concludes by identifying three key characteristics of this labor market: (i) its heterogeneity, (ii) an internal logic that amplifies and naturalizes precariousness, and (iii) its capacity to offer possibilities for rapid job insertion and precarious social inclusion.

## 1 Introduction

In Chile, as in other Latin American countries, a growing expansion of digital labor platforms has been observed since 2010 [Arriagada et al., 2023; Fairwork, 2024; Superior Labor Council, 2024, 2025; Morris, 2021; Gontero and Ravest, 2024; International Labour Organization (ILO), 2024b]. Recent figures from 2024 indicate that around 280,000 people in the country—representing 3.1% of the employed population—work and generate income using a mobile application or web platform (Gontero and Ravest, 2024; Superior Labor Council, 2024, 2025). That number of workers represents a 200% growth compared to 2020.

The objective of this article is to contribute to the understanding of these new labor markets of digital labor platforms in Chile, based on the reconstruction of the case of delivery and ride-hailing platforms. Drawing on key elements of Grimshaw's “New labor market segmentation approach” framework (Grimshaw et al., 2017; Fernández-Huerga, 2010), and based on three empirical studies conducted between 2019 and 2025, this article offers a multidimensional analysis of the particularities of this labor market by examining three axes: (i) platform companies, (ii) State labor regulations, and (iii) workers. The analysis incorporates a diachronic perspective that highlights some central milestones in the development of this labor market in Chile between 2010 and 2025, accounting for its dynamism and transformation. Likewise, a perspective is incorporated that accounts for the role of different actors - business actors, State actors (legislative, executive, and judicial branches) and worker organizations- in shaping the labor market of digital work platforms in Chile.

The analysis we present is situated within and engages with current debates in the sociology of work in Latin America regarding the strong expansion of digital labor platforms in the region (Abílio, 2023; Filgueiras and Antunes, 2020; Haidar and Keune, 2021; Palermo et al., 2025; Stecher and Morales, 2024; Veras de Oliveira and Bridi, 2023; Zukerfeld et al., 2024). From this field of discussions, we highlight three main theses, which are central to understanding the labor market of digital platforms in Chile.

Firstly, extensive documentation shows the substantial expansion of this labor market across Latin America beginning in the 2010s, a trend that experienced significant acceleration throughout the COVID-19 pandemic. During this period, the economic crisis, unemployment, and lockdown measures drove growth in the number of workers seeking to generate income through platforms, concurrent with a marked increase in e-commerce and consumer adoption of digital platforms [International Labour Organization (ILO), 2024b; Stecher and Morales, 2024; Veras de Oliveira and Bridi, 2023]. This is a labor market with low access barriers, which offers quick income possibilities to very diverse worker profiles (immigrants, women, students, youth from popular sectors, unemployed middle classes, etc.) [Gontero and Ravest, 2024; International Labour Organization (ILO), 2024b]. What was once considered a novel phenomenon has now stabilized as an important and permanent segment of the region's labor markets, with a workforce for which platform work is predominantly (61%) their main source of income and which dedicates on average over 30 weekly hours (32.6 h) to this activity [International Labour Organization (ILO), 2024b]. This is an internally differentiated labor market, where location-based platforms (delivery, transport, domestic service, etc.) and web-based platforms (microtasks, freelance, etc.) coexist, encompassing national companies, Latin American multinationals, and global multinationals (Gontero and Ravest, 2024; Woodcock and Graham, 2020), all of which express broader structural and global trends toward digitalization, platformization, and informalization of work characteristic of contemporary capitalism (Abílio, 2023; Leite et al., 2024; Mezzadra and Neilson, 2024; Srnicek, 2017; Vallas and Schor, 2020; Zukerfeld et al., 2024).

Secondly, Latin American Social Sciences scholarship has shown how the expansion of these platforms in the last decade is grounded in, while simultaneously amplifying, the principles of neoliberal reforms and productive flexibility logics implemented since the 1980s in the region: The intensification of employment precariousness logics under the figure of platforms workers as independent contractors without access to social protection and collective labor rights; the presence of a business model that takes to the extreme the initial logics of productive decentralization and fissurization of work, now reduced to a sum of specific tasks that are paid only once performed; the maintenance of strict disciplining and managerial control over work processes based now on algorithmic management mechanisms; as well as the strengthening of highly individualized imaginaries and ideals of the neoliberal labor subject based on the values of entrepreneurship and autonomy (Bensusán and Santos, 2021; Hidalgo and Salazar, 2020; Salas et al., 2024; Filgueiras and Antunes, 2020; Stecher and Morales, 2024; Veras de Oliveira and Bridi, 2023).

Thirdly, recent research has highlighted the particularities of the expansion of digital labor platforms in Latin America, in contrast to the situation in the global north. In this region, the platforms are inserted in labor markets where informal employment rates currently average 48% [International Labour Organization (ILO), 2024b], where unprotected, unstable and precarious employment has been—since the beginning of capitalist modernization—the historical majority norm, where many of the formal jobs are equally unstable, and characterized by low salaries, authoritarian management, limited possibilities for development and various forms of discrimination, and where the neoliberal shift has had a substantial impact on the level of privatization and commodification of access to social protections and rights (Abílio, 2023; Salas et al., 2024; Stecher and Morales, 2024). In this context, digital labor platforms have found a propitious terrain in which to expand in Latin America in the last decade, characterized by a strong economic stagnation after the end of the commodities boom (2003–2011) and deepened by the severe economic crisis and job destruction during the pandemic. The expansion of digital labor platforms in Latin America, as pointed out by Veras de Oliveira and Bridi (2023), has reinforced and legitimized historical and previous trends of informal and unprotected employment, but reconfiguring them under new digital technological mediations, new entrepreneurialized symbolic frameworks and based on their direct articulation with large multinational companies inserted in global circuits of accumulation (Manky et al., 2024; Veras de Oliveira and Bridi, 2023).

This article seeks to contribute—through the case of delivery and ride-hailing platforms in Chile—to the field of discussion on digital platform labor markets in Latin America. The specific relevance and contribution of the article is based on two main points. First, the particularity of the Chilean case, considering its pioneering and emblematic character in terms of the early adoption of neoliberal reforms and the radical nature with which they transformed the worlds of work and social life since the late 1970s (Stecher and Sisto, 2019); reforms whose logic operated as a condition of possibility for the organizational and business model of digital labor platforms. Additionally, Chile represents a case of interest because, in the last 3 years, different laws and regulations enacted by the State have impacted the labor market for delivery and ride-hailing platforms (Martín-Caballero, 2025). Secondly, the article constitutes a contribution to the debate on the labor markets of digital labor platforms by offering an in-depth and complex reconstruction of the Chilean case based on the analysis of diverse primary and secondary sources, from a temporal perspective and discussing the role of different actors, and addressing three dimensions: (i) a characterization of the *companies* in the sector, their business model and the profile of the workforce they recruit; (ii) a discussion of the *State* labor regulations and employment status of platform workers, with a focus on the recent implementation of law 21.431; and (iii) a description of *workers* that considers everyday workplace relationships and forms of collective action and organization.

## 2 Data collection methods and analysis techniques

To address the article's central aim concerning the principal characteristics of the digital delivery and ride-hailing platform labor market in Chile, a corpus of empirical material produced by the authors within the framework of three research projects on digital work platforms over the past 6 years (2019–2025) was analyzed. All studies conducted comprehensive reconstructions of company operations, state labor regulations, working and employment conditions, forms of workers' associativity and collective action, and workers' experiences and subjectivities.

The first project was based on a qualitative ethnographic design and included different data production strategies: documentary analysis of primary sources (such as newspapers, legislative debate records, company websites, social networks of workers' groups and associations), four ethnographic interviews focused on the description of the operation of the applications (Apps), 30 in-depth interviews of 90 min with 14 delivery workers and 16 ride-hailing drivers (23 men and 7 women) from 3 cities in Chile, and participation in two assemblies of delivery workers.

The second study had a qualitative design based on a narrative approach and focused on delivery workers. It contemplated narrative interviews with 21 workers in the city of Santiago (11 men and 10 women) and the documentary analysis of the narratives and meanings about delivery work present in advertising videos of 3 delivery platforms in Latin America—two of them: Uber and Pedidos Ya—in Chile.

From the third research, still in progress, and qualitative and based on a narrative approach, the following information production was contemplated for the following article: 12 semi-structured interviews to key informants -companies, academics, leaders of organizations of workers and State actors-; 19 interviews to workers, 13 delivery workers and six ride-hailing drivers, 16 men and three women, from the cities of Santiago and Concepción; 4 visits and field notes in places enabled to rest for delivery workers in the city of Santiago; documentary analysis of the web pages of 9 delivery and ride-hailing digital platforms operating in Chile; and review of secondary sources including reports from national and international organizations regarding platform companies, labor market dynamics in this sector, and employment and working conditions.

The 70 interviews with workers allowed us to cover a wide range of variability concerning those dimensions of particular importance for the sector referred to in the specialized literature: age, immigration status or not, weekly hours dedicated to the platform, membership in workers' associations, care responsibilities, primary or secondary source of income, and length of service in the sector.

For this article, the empirical corpus and pre-analysis documents previously elaborated were subjected to a thematic content analysis guided by the guidelines of Braun and Clarke (2024). This method is based on systematic and reflexive data coding processes to progressively develop and identify themes and sub-themes relevant to the research question and for the theoretical framework of the study. For the present article, the data were interrogated to reconstruct the main characteristics of the labor market of digital delivery and ride-hailing platforms in Chile, considering the three dimensions of analysis outlined above.

All the studies were approved by the research ethics committees of the principal investigator's universities, and informed consent protocols were used to guarantee the confidentiality and anonymity of the information provided to the participants.

## 3 Results

### 3.1 Companies: business model and the profile of the workforce

#### 3.1.1 Companies and their business models

Digital ride-hailing and delivery platforms have undergone a gradual insertion process into the Chilean economy and labor market since 2011, beginning with the entry of the first delivery company, PedidosYa, followed by the first ride-hailing company, Cabify, in 2012. Since then, particularly with the entry of Uber (ride-hailing) in 2014 and Uber Eats (delivery) in 2017, this platform-based delivery and ride-hailing sector has become an essential segment of service sector economic activity and a labor market that provides regular income to a growing number of workers across the country's main cities.

The platform companies in Chile share similar business models and organizational structures among themselves, characteristics that reflect global operational patterns typical of delivery and ride-hailing digital labor platforms [Gandini, 2018; International Labour Organization (ILO), 2021; Vallas and Schor, 2020]. This approach is based on digital intermediation between labor demand and supply for passenger transportation services or food and product delivery. Through smartphone applications that function as digital infrastructures, companies facilitate and regulate interactions between service demanders (clients), intermediaries (platform companies), workers, and—in the case of delivery—restaurants and commercial establishments, generating profits by charging clients, and/or workers, and/or restaurants or stores for each completed task or service (Vallas, 2018). The model relies on the collection and management of large volumes of data and the deployment of algorithmic control mechanisms through which tasks are assigned, rates are established, customer evaluation systems are administered, and incentives and penalties are implemented, among other functions (Gandini, 2018; Kellogg et al., 2020). The model assumes the classification of workers as independent contractors who must bear the risks and costs of their work, receive payment per completed task, generally lack full access to social protections and labor rights, maintain certain flexibility margins to define schedules and work locations across different platforms, and are encouraged to identify themselves as entrepreneurs and company partners (Gandini, 2018; Nicoli and Paltrinieri, 2019; Vallas and Schor, 2020).

Within this common framework, the conducted analysis identified certain variations between platform types and among companies within the same platform category, including aspects such as registration requirements, whether or not there are visual recognition mechanisms from the app when activating the account, fare criteria and rates, whether or not a percentage of the service is charged to workers, and if so, the amount of that percentage, and whether or not there is a requirement for workers to define schedules and service areas in advance in the app. As one interviewed driver noted, illustrating these variations: “*Uber takes a 50% commission of what you pay. DIDI 30%, Cabify is 25%, and InDriver in some areas is 0% and in others 12%.”* Another driver comments about the InDriver platform, which would be very insecure since there is very little control in the registration of drivers and passengers: “*InDriver is super insecure and you have to choose the trips, but very carefully, especially me who works at night, (…) Because it doesn't ask for any kind of registration to make you an account, eh… you are supposed to upload a picture of yourself, but you see people with pictures of kittens, pictures of Hello Kitty…”* (Ride-Hailing platform, driver, 37 years). In delivery platforms, the difference between the Uber Eats system and Pedidos Ya stands out, with the latter company requiring delivery workers to register in advance for specific schedules and service areas. As one interviewee tells us: “…*Pedidos Ya makes you stick to a schedule and makes you work in a particular area (…) With Uber [on the other hand] I had flexible hours where I could connect anywhere, anytime. I didn't have that rule of having to go to a specific place to connect, or a specific time to connect*” (Delivery worker, 30 years). While giving a detailed account of these variations between platform companies is not the focus of this article, it is important to note—based on these examples—how within a similar business and operational model, these differentiations exist within this sector.

Another characteristic of the digital labor platform economic sector is its oligopolistic character, with very few companies concentrating large market shares. In the case of delivery services, Uber Eats, PedidosYa, and Rappi are the main players, each with between 7,000 and 10,000 active delivery workers and 15,000 associated businesses (restaurants, stores, pharmacies, etc.) with whom they establish diverse contractual arrangements and from whom they collect commission percentages on each transaction and delivery service (La Tercera, 2024). In the case of ride-hailing platforms, Uber is the main player, alongside Didi and Cabify (National Productivity Commission (CNP) and Fundación Chile, 2018). These are companies—particularly in the delivery sector—with substantial investment and expansion plans in Chile in recent years, with amounts reaching $32 million for Rappi and $40 million for PedidosYa by 2025. In general terms, platform companies in Chile -except for Cabify and to a lesser extent Pedidos Ya- have consistently obtained very low scores in Fairwork's studies on compliance with minimum standards of fair labor (payments, conditions, contracts, management, and representation) (Fairwork, 2024).

The Table 1 summarizes the main ride-hailing and delivery platforms operating in Chile:

**Table 1:** Main ride-hailing and delivery platforms operating in Chile.

**Platform type**	**Company**	**Year of creation**	**Year of entry to Chile**	**Number of registered workers (not necessarily active)**	**Fairwork score**	**Entry requirements**
Ride-hailing platforms	Cabify	2011	2012	There are no precise figures for each platform. An estimated 105,000 registered drivers are on ride-hailing platforms	5/10	Rigorous. Professional driver's license required
	Uber	2009	2014		0	Moderately rigorous. Does not require professional driver's license required
	DiDi	2012	2018		0	
	InDrive	2012	2019		0	Low stringency. Request professional driver's license and years of driving experience
	UpGirl	2020	2020	700 female drivers registered (2022)	Not considered	Rigorous, among which being a woman stand out.
Delivery platforms	PedidosYa	2009	2011	There are no precise figures for each platform. An estimated 300,000 registered workers are on delivery platforms (not necessarily active)	2/10	Low rigorousness, among which the following stand out: foreigner's identity card or document, valid license, vehicle registration certificate
	UberEats	2014	2017		0	Low rigorousness, among which the following stand out: valid license, vehicle registration certificate, etc.
	Rappi	2015	2018		0	Low stringency, among which the following stand out: ID card, valid license, vehicle registration, bank account, etc.
	Justo	2019	2019		1/10	Low rigorousness, among which the following stand out: identity card or foreigner's document, valid license, bank account, etc.

The majority of these companies are regional or global multinationals that operate through transnational and very standardized management models, maintain a presence in major urban centers, and employ substantial numbers of professionals, managers, and support technicians in their administrative offices. These companies deploy remarkably homogeneous discourses regarding their economic role within the national context. Through their websites, advertising campaigns, and public statements by corporate representatives, platform companies emphasize their importance and social contribution by generating novel forms of technologically mediated employment—forms that, they argue, should not be evaluated within traditional labor relations frameworks. According to this narrative, these employment arrangements offer significant benefits due to their inherent flexibility and autonomy, low barriers to entry, hourly compensation rates that exceed minimum wage standards, and substantial social utility by delivering faster, more available and more affordable services that improve people's lives. This discourse strategically highlights the appeal and social value of platform employment models while simultaneously obscuring the inherent risks and precarious nature of such work arrangements. Consequently, it legitimizes a business model predicated on the independent contractor framework, positioning workers as autonomous entrepreneurial agents rather than traditional employees.

As one interviewed key informant (KI)—a manager from one of the delivery platform companies—points out:

… Delivery, in reality, is a service that facilitates people's lives in the cities, that was the origin, the philosophy behind the Delivery companies. (….) this was a phenomenon that happened all over the world.(…) So in reality this way of working is very attractive for people in general for, for two main reasons, the first one is because the earnings are good, ok? (…) Delivery drivers earn, taking out their costs, taking out their own operating costs, let's say, they earn above the minimum wage, eh, so it becomes attractive in terms of income and on the other hand, it also becomes attractive in terms of flexibility. Why? Because the model is a model in which the delivery drivers choose when to connect, choose when to disconnect, choose when they want to provide services, where they want to provide their services, and that gives them a lot of flexibility compared to other types of jobs (KI 3).

It is also important to note that this is a dynamic and highly competitive business sector where there have been purchases of companies by large multinationals (for example Easy Taxi was incorporated into the Cabify platform in 2019, and Uber bought the Chilean supermarket shopping and delivery company Cornershop on 2021), as well as the exit of companies from the country (for example the Spanish Glovo that closed its operations in Chile in 2019 or the Greek transport company Beat that operated until 2022). This is a sector with high levels of concentration among large multinational corporate actors, which deploy high investment in technology and operate at large scales, and where it is very difficult for new and smaller companies to enter and compete, unless they offer a very specific and different service. As one key informant explained, a manager of a small Chilean ride-hailing platform focused on services not addressed by large companies.

Because Uber, with its model—or all these Uber-type platforms—precisely requires so much scale, having 200,000 drivers and 2 million passengers, because it's an on-demand, occasional model, where you think: “ah, I want a ride now, it just occurred to me,” and there has to be an available driver nearby (…) Uber isn't just a product, it's not just the app, today they have data management, data analysis, innovation in terms of dynamic pricing, how we tell the driver where to go, how we activate facial recognition every so often. That's millions and millions and millions of dollars. (…) I was able to compete [only] because I compete in a niche where I have a different value proposition, where I offer a different service…. If I set out to make a Chilean Uber, I don't compete, I can't compete (…) if you set out, without any differential attribute, to be just another one, you're lost. Because the technology [costs] are impossible (KI 4).

It is interesting to note that this tendency toward market concentration in large multinational platforms has produced a homogenization at the level of business and operational models of companies. In Chile, in this regard, the emblematic case was UBER's acquisition in 2021 of the Chilean platform Cornershop, which had been created in 2015. In its first 2 years, Cornershop operated with a model in which it hired shoppers/delivery workers under employment contracts, who underwent rigorous selection and training processes and later formed Chile's first platform workers' union in 2017. The former Cornershop workers interviewed—many of them now working for UBER Eats—express nostalgia for the former company, which allowed them to develop skills, obtain recognition from customers who highly valued the service, to have (human) support from the company always available for any questions or contingencies, and have a clear, predictable, and transparent payment mechanism. UBER's acquisition meant the end of early Cornershop's model and the shift to the standard platform model with independent workers, low barriers to entry, unclear and changing criteria for fare setting, limited training, opaque evaluation systems and limited opportunities for career development and recognition.

The growth of platform companies in Chile has had as a condition of possibility—given their business and operational model—the continuous availability of a broad workforce. Our analysis of primary and secondary sources allowed us to identify 4 structural aspects underlying this availability of workers for platforms.

First, the substantial increase of Venezuelan immigration to Chile, whose number grew from 55,000 immigrants in 2015 to 728,000 in 2023, currently representing 38% of the immigrant population in Chile, and constituting a massive, highly skilled labor force that found in the platforms -especially in the delivery platforms- an expeditious and low-barrier way to enter the world of work and generate income, and even with the possibility -by using borrowed or falsified accounts and avoiding police checks on licenses- of working without having to regularize their legal status or obtain a national driver's license.

Second, there is a stagnation of the Chilean economy that went from growing 6.1% in the 1990s, to 4.2% between 2000 and 2009, to reach 1.9% in the period 2014–2023. This more structural trend, to which was added the economic impact of the social outbreak (2019) and the pandemic (2020–2021), shows a fragile labor market where many of our interviewees were middle class professionals, over 40 years old, who had lost their jobs and, faced with the impossibility of reinserting themselves into the labor market, ended up working on digital platforms, in many cases using their compensation to buy a car. Unlike the group of Venezuelan immigrants, this profile tends to be inserted primarily in ride-hailing companies, or delivery using a car and focusing on supermarket shopping.

Third, the conditions in the formal sector of the economy of low salaries, rigid working hours, neotaylorization of work processes and authoritarian relationship patterns, especially in low-skilled jobs in the service sector, sometimes favor the search for alternatives in the segment of platform companies, whose greater flexibility in working hours, less direct subordination to despotic bosses and possibilities of adjusting remuneration to needs, based on the increase in working hours, is attractive. This phenomenon can be seen in some cases of low-skilled national workers or higher-skilled foreign workers who actively choose to move from salaried jobs in the formal sector to the platforms (Morales and Stecher, 2023).

Fourth, the platforms are attractive due to their flexible working hours for young students who work in specific limited shifts, for workers with stable jobs who seek to supplement their income with some hours working on the platforms at the end of their workday or on weekends, or for people -especially women- with domestic and care tasks who, in the context of a strong privatization and commodification of social rights in the country, must support and self-manage these tasks based on individual and/or family arrangements.

Some testimonies allow us to illustrate these structural dynamics of the world of work in Chile that help understand the capacity that platform companies have had to attract a large contingent of workforce:

“I arrived—by saying—today to Chile, and the next day I was already working on a motorcycle, with an account that they lent me, it was not mine at that time because I had just arrived, I had no papers or anything and I was already working and I adapted quickly (…) (…) and then [some of the companies] were accepting delivery drivers without much paperwork, they accepted you with your passport, with your Venezuelan license (…) they practically accepted everyone, something like that (laughs)” (Male delivery worker, Immigrant, 29 years old).

…in this country, after the age of fifty, you are already old, therefore, you are absolutely discriminated against, they throw you out of the jobs, out of management positions and all that, they just eliminate you, and for the money they pay you they hire two guys just out of university… (…) those who arrived in the pandemic [to the platform], the majority are all professionals, older than fifty, who with the pandemic were dismissed. (…) And they were never able to find work again, so they are captive just like me, because at my age, at fifty-7 years old, I can't find a job anywhere (…).” (Female delivery worker, Chilean, 57).

In this capacity to mobilize a heterogeneous workforce, taking advantage of the fragilities of the formal labor market, the precariousness of immigration processes or the weakening of institutional supports for social protection; together with the possibility of managing and controlling—from a distance—their work processes through algorithmic management mechanisms; maintaining their status as independent workers; offering them the ideal of entrepreneurship and autonomy; and expanding their daily presence in the cultural practices of society and customer demand; platform companies in Chile have managed to consolidate their position as relevant players in the economy.

#### 3.1.2 The profile of the workforce

Delving deeper into the profile of the workforce that works in platform companies in Chile, it is possible to highlight the following data and characteristics of delivery and ride-hailing workers.

Instituto Nacional de Estadísticas (INE) (2022) (National Statistics Institute) data indicate that around 280,000 people -representing 3.1% of the employed- declare that they work, as their first or second occupation, using some mobile application or WEB platform (Superior Labor Council, 2024). This figure also includes people who use platforms such as WhatsApp, Instagram, or Facebook to sell products, which, strictly speaking, are not part of the labor market of digital labor platforms. Of this global universe, the data report 52% male workers and 48% female workers, as well as 62% informal workers and 38% with some degree of formalization (Superior Labor Council, 2024).

Focusing specifically on the delivery and ride-hailing platforms on which this article is centered, and which are those regulated in Chile by the new law 21.341, the official (Instituto Nacional de Estadísticas (INE), 2022) data report that 80,000 people, 0.8% of the employed, are employed in these platforms as a first or second occupation. As of February 2025, 68% of this total corresponds to informal workers (Superior Labor Council, 2025). Other recent estimates indicate that between 100,000 and 120,000 people would obtain income -not necessarily as first or second occupation- from such platforms (Centro de Políticas Públicas UC (CPP-UC), 2023; Servicio de Impuestos Internos (SII), 2025), which would account for up to 1.4% of the total number of employed people in the country. Of these workers, 62.5% work on ride-hailing platforms, 27.5% work on delivery platforms, and 10% combine both platforms (Superior Labor Council, 2024). Likewise, the companies' data refer to the number of workers with registered accounts, not necessarily active or used, which are around 405,000 people, representing around 4.5% of the employed.

Going deeper into the composition of the labor force of delivery and ride-hailing platforms in Chile and its socio-demographic profile, it is possible to point out that it is a strongly masculinized labor market, with 85% of workers reported to be men; likewise, there is a high rate of foreign workers, reaching 41.7%, and in line with the reality of other countries in the region, there is a profile of young workers, with 70% of workers under 40 years of age. It should also be noted that this is a workforce with high levels of qualification, with 30% having completed higher education and over 94% having completed secondary education (Superior Labor Council, 2024). This is a platform worker profile very similar to that reported for other Latin American countries [International Labour Organization (ILO), 2024a,b; Stecher and Morales, 2024]. It must be considered, however, that these averages obscure some important differences between delivery and ride-hailing platforms, which we present below.

Regarding ride-hailing drivers, it is possible to estimate a mostly national workforce, with only 20% of immigrant workers, with an average age of 38 years (Superior Labor Council, 2024; Centro de Políticas Públicas UC (CPP-UC), 2023). This data highlights the relative nature of the low entry barriers mentioned earlier. Although the requirements are lower compared to formal employment, delivery and ride-hailing platforms require each worker to be equipped with a vehicle—whether a bicycle, motorcycle, or car—which entails an initial investment or personal asset in all cases. However, when a car is required, as is the case with ride-hailing platforms, this investment is considerably higher, constituting a significant entry barrier to the workforce, with particular relevance for immigrant workers. The surveys estimate that for 60% of app drivers, this job represents their only or main source of income, with an average of about 17 h per week. Nevertheless, 50% work around 42 h per week, and 25% work approximately 54 h per week. For many drivers, platform work involves operating from Monday to Sunday, sometimes in shifts lasting up to 12 continuous hours (FLACSO, 2022, 2023). Field findings indicate that ride-hailing drivers earn slightly more than those in delivery platforms, reaching an income equivalent to or somewhat higher than two minimum wages for 44 or more hours of work, including tips and after deducting gasoline costs. Finally, drivers usually operate across multiple platforms, the most popular being Uber, Cabify, and recently DiDi, with an estimated 10% participating in trade unions or sector associations (Centro de Políticas Públicas UC (CPP-UC), 2023).

With respect to the delivery platforms, there is a higher percentage of immigrant workers—over 70%—and a lower average age of workers, with an average age of 32. On average, delivery workers work around 20 hours per week (Centro de Políticas Públicas UC (CPP-UC), 2023), 50% dedicate more than 42 hs, and 25% reach 60 h per week, which implies that many work from Monday to Sunday and in continuous 12-h workdays (Superior Labor Council, 2024; FLACSO, 2023). It is estimated that for 70% of the people who work in delivery, this type of work constitutes their only or primary source of income, which shows processes of “professionalization” and “stabilization” in these activities, different from the context of some countries in the global north where the use of platforms as complementary sources of income predominates. In terms of income, and for those workers who work more than 50 h per week, it is possible to reach incomes equivalent to between 1.5 and 2 minimum wages. Finally, like drivers, delivery workers tend to use more than two platforms, the most popular being Uber Eats, Rappi, and PedidosYa. Delivery workers' participation in collective organizations or trade unions is estimated at 5% (Centro de Políticas Públicas UC (CPP-UC), 2023).

### 3.2 The state: regulatory framework, labor protection, and social security

As at the global level, both in Chile and in Latin America, there has been a broad debate on the employment status of platform workers and the best way to regulate and grant protections to such forms of work, with different positions ranging from the urgency of recognizing the employment relationship given the multiple signs of subordination and dependence, to those who advocate maintaining the status of independent worker and deny the employer character of the platforms and their responsibilities in guaranteeing individual and collective rights to workers (Martín-Caballero, 2025; Palomo Vélez et al., 2022; Stecher and Morales, 2024). Within this latter pole, some positions recognize the importance of maintaining the status of independent workers (self-employed workers), or creating a new intermediate status, to generate specific mechanisms that offer platform workers access to some levels of social security, labor protection and —to a lesser extent— collective rights (International Labour Organization (ILO), 2024a).

Another more specific regulatory debate, limited to ride-hailing platforms, concerns how to regulate these services, which usually do not comply with the requirements and standards of traditional taxi or similar services. This regulation refers not so much to the employment status and employment contract as to the regulation of an economic sector and the requirements -for drivers, vehicles, platform companies- to participate in it. These are sectoral requirements that also affect the configuration of the labor market of platforms.

After long processes of debates and public dispute, and extensive legislative procedures of more than 5 years, Chile has passed laws for both issues. Law 21.431 approved in March 2022 and in force since September of the same year, which “*modifies the Labor Code regulating the contract of workers of digital service platform companies*”; and Law 21.55, which “*regulates paid passenger transportation apps and the services provided through them*” approved in April 2023, but with entry into force only during 2025 due to the significant delay in the approval of its implementing regulations.

#### 3.2.1 Law 21.431: regulation of work contracts for digital service platform company workers

Chile was the first Latin American country to approve a law that regulates work contracts for location-based digital platform workers. Law 21.431, approved by Congress in March 2022 and implemented since September of the same year, modifies the Labor Code by introducing a new chapter specifically referring to work through location-based digital service platforms. It is a hybrid law that does not settle a priori the employment status of platform workers. The law -as we will see- establishes the possibility that workers can be considered as “dependent platform workers” or “independent platform workers,” granting the latter a set of labor protections and access to individual and collective rights.

Until the approval of this law, and since the beginning of their operations, most of the delivery and ride-hailing platforms operated under a logic of informal, unregistered employment. Instead of formal contracts, workers were subject only to a digital acceptance of terms and conditions, without any labor or social protections (Asenjo and Coddou, 2021; Fairwork, 2021).

Before detailing the contents of the law, it is important to highlight some of the factors that explain why Chile -one of the Latin American countries that most radically experienced processes of neoliberalization, flexibilization and deregulation of employment- has been a pioneer nation in establishing this regulation.

First, it is important to note that the law's discussion process took place between May 2020 and March 2022, in the midst of the pandemic context, where quarantine measures made visible both the social utility of the service provided by delivery platform workers, as well as the precarious conditions (accident risks, contagion risks, absence of protections) under which they carried out their work (Gutiérrez, 2025). Likewise, the economic crisis and job destruction in the pandemic context made more visible in public debate the role of platforms as alternative spaces for labor insertion and income generation.

Second, it should be noted that the discussion of the law takes place in a context of strong growth of organizations and protest actions by delivery workers, which in Chile reached their peak precisely in 2020 with 5 public protests (Gutiérrez, 2025; Gutiérrez and Atzeni, 2022). As we will explore further ahead and as in other Latin American countries, the context of precariousness and vulnerability of the pandemic was the moment of greatest strength for organizations and collective action by platform workers. One line of action and protest involved submitting claims to the courts to have the employment relationship of subordination and dependence recognized. This led to landmark rulings, such as the decision on October 5, 2020, in which a court in the city of Concepción recognized the existence of an employment relationship between a delivery worker and the company Pedidos Ya, and ruled that his dismissal was unjustified (RIT M-724-2020). The collective organization and protests of workers, as well as the legal demands and favorable rulings that questioned the non-recognition of the employment relationship, also influenced the legislative debate, leading different actors -even those initially reluctant to any regulation of platforms- to recognize the convenience of establishing some type of regulatory framework for these forms of work. In this way, achieving a regulation that would avoid permanent judicialization, and that, at the same time, maintained and legitimized the figure of the independent worker -granting him from the same labor code some protections and rights- appeared as a hybrid formula that was viewed favorably by company representatives and certain political groups that participated in the legislative debate. As a labor law expert tells us:

And also, this [the approval of law 21.431] had a reason that I would say circumstantial, which were the court rulings. Judgments began to appear, particularly the one in Concepción, which began to arrive, and which was ratified by the Court of Appeals, which was a warning for the companies, fundamentally in the sense of saying that this could not continue to happen on a massive scale and consolidate, in short, a labor thesis in the courts (…) Therefore, what ended up appearing was a hybrid norm, as I pointed out, also very exotic for our legal formula. For the first time a figure of an independent worker is incorporated within the labor code, which was very rare, let's say. Because the focus was on dependent workers and now it seems this other figure in between, which has been complicated in its application” (KI 1).

Thirdly, the Chilean political context and the dynamics of the legislative debate help explain why the law that was ultimately approved represented a compromise between the positions of two earlier bills on the same issue. On the one hand, parliamentarians from the Frente Amplio (left-wing opposition at the time), together with delivery workers' organizations, drafted in March 2019 a bill called “*My Boss is an App”* (Bulletin No. 12.475-13), which recognized the labor nature of the relationship between the company and its workers and established regulation for payment of the passive workday (the time during which the worker is at the company's disposal). On the other hand, while the opposition proposal was still being processed, the government (a center-right political coalition) introduced in May 2019 a labor modernization bill that included several flexibilization measures and excluded workers providing services through digital platforms from labor legislation (Bulletin No. 12.618-13), receiving the support of the companies. In a socio-political context (2019–2021) characterized both by strong citizen criticism of social precariousness and the neoliberal model—linked to the October 2019 social unrest and the first constituent process—and by the presence of a center-right government with a strong pro-business orientation and a congress without a clear majority for any political bloc, the third bill, given its intermediate and hybrid nature, emerged as a consensus instrument among the various actors (parliamentarians, companies, workers, academics, etc.). It was approved in March 2022 and came into force in September of the same year (Leyton and Azócar, 2022). It should be noted, as part of the context that favored the aforementioned “mediator” project, that significant differences existed among the workers' own organizations. While some delivery worker organizations supported the first legislative initiative promoting formal dependent employment status (“*My Boss is an App”*), driver organizations generally advocated for legislation that—while granting access to certain specific rights and protections—would preserve their status as independent contractors. As noted by a union leader representing app drivers who participated in the legislative debate on the law (KI2).

There was [the project] “My Boss is an APP” [pro-laborization project], right? And there was “Basic Guarantees” [project]. We supported “Basic Guarantees”… and it gave us the status of “independence,” which is what we were accustomed to. I have been a driver for seven years—something I hadn't mentioned before. Having been a driver for 7 years now, you get used to working freely, and with ‘My Boss is an APP” you would be confined to a specific schedule—forget it!. We fought and fought for independence; all the campaigns that were carried out were for independence.

Turning to the actual content of Law No. 21.431 that was finally approved, it should be noted that while this law aims to regulate relationships between workers and digital service platform companies, it does not determine the type of contractual relationship between companies and workers. The law leaves open the possibility—depending on agreement between the parties—that such relationships may be either dependent (with employment contract signing) or independent (with service provision contract signing), which would depend on whether the presence (or absence) of subordination and dependency relationships established in the labor code can be proven.

The law establishes a comprehensive set of new obligations for platform companies, the vast majority of which apply to both dependent and independent workers. Some of these include: clear contract formulation, establishment of a communication channel with companies, maximum working time of 12 h of continuous connection, explicit mechanisms to set service prices or rates, rules for protecting workers' personal data, labor safety training, provision of individual protection equipment (helmets, knee pads, etc.), guarantee of minimum hourly wage, damage insurance covering personal property, establishment of contract termination grounds and communication methods, proportional compensation not less than the minimum monthly income increased by 20%, duty to inform workers prior to service acceptance of service details (destination address, payment method, etc.), and respect for workers' constitutional guarantees. The law also establishes that the programming of algorithms used by companies may be subject to oversight and must avoid any form of discrimination. Furthermore, regarding collective rights, the law enshrines platform workers' rights—both dependent and independent—to form labor unions and engage in collective bargaining. However, in the case of unions with—or of—independent workers, such negotiation takes place under “non-regulated” processes (Article 314 of the Labor Code), which establishes that negotiation is voluntary for both parties and does not grant workers and leaders the right to strike or job protection (Leyton García and Azócar, 2022; Gutiérrez, 2025; Superior Labor Council, 2024).

Alongside these labor protections and access to individual and collective rights, the law requires—for independent platform workers with service provision contracts—the issuance of a fee invoice for the services performed, which constitutes a tax document associated with the payment of a tax (14.5%) that is reported to the Internal Revenue Service and results in the formalization of labor activity. In turn, based on this issuance of fee invoices and tax payment, independent platform workers gain access to social protection coverage under another law—Law 21,133 of 2019 that “*Modifies the rules for incorporating independent workers into social protection regimes”*. This includes access to Disability and Survivor Insurance; Social Insurance against Work Accident Risks and Occupational Diseases under Law No. 16.744; Accompaniment Insurance for Children with Serious Health Conditions under Law No. 21.063 (Sanna Law). Likewise, this mechanism allows for the payment of health contributions—which provides access to the right to medical leave—and contributions for pensions (old age, disability, death). This process of formalization and access to social protection for independent platform workers was expanded and strengthened starting in September 2023 with the signing of Resolution 132 of the Internal Revenue Service (SII), which established that it was the platform companies themselves—and no longer each individual worker—who had to withhold workers' taxes monthly and report and pay them monthly to the SII (Ministry of Labor and Social Welfare (MTPS), 2025). Recent SII reports show a fourfold increase in the number of fee invoices issued between February 2024 (40,000) and August 2024 (180,000), marking a positive trend toward formalization and access to social protection for independent workers at platform companies (Ministry of Labor and Social Welfare (MTPS), 2025).

Regarding the implementation of the law in its first 3 years, the following aspects are worth highlighting:

First, the Ministry of Labor has established various social dialogue mechanisms, organizing roundtables with platform companies, worker organizations, and sectoral business associations (ACHIPLAM and ALIANZA IN CHILE) (Superior Labor Council, 2024, 2025; KI 10, KI 11). This dialogue has been informed by annual reports on the implementation and application of the law prepared by the Superior Labor Council (2024, 2025), which were mandated by the law itself for the first 3 years of its validity. A important result of the law is that it has legitimized in public debate, and for the different actors in the platform labor market, the fact that this is a sector that must be regulated in some way, and whose workers must be subject to protections and access to rights through labor institutions.

Second, it is possible to observe that the law has naturalized and legitimized the figure of the independent platform worker, who—while having obtained certain rights and protections—has not been able to access formal employment contracts from companies, even in cases where signs of an employment relationship (subordination and dependence) are evident. Although the law offers both possibilities and the Labor Directorate issued a ruling (No. 1831/39) in October 2022 identifying criteria to distinguish between both contractual figures—a ruling that was rejected and challenged in court by companies until it achieved final approval by the Supreme Court—in practice, companies have made massive and exclusive use of the independent worker figure, given the existing power asymmetries between them and workers. This has produced a legitimization of this contractual form, which had previously been questioned by court rulings in 2020. The law, in this way, by validating the independent worker figure—without assuming the presumption of employment relationship as a starting point, as in other international legislation—strongly diminished the possibility of filing lawsuits against companies in courts. Labor law experts are generally critical of these consequences of the law. They express concerns both regarding digital work platforms and the possibility that the figure of formalized independent workers becomes legitimized and expands to other labor spaces, replacing employment contracts based on dependence and subordination and generating widespread precarizing effects across the labor market (Ugarte, 2020; Leyton and Azócar, 2022; Palomo Vélez et al., 2022).

Third, it is possible to point out—as two key state informants interviewed indicated (KI 9, KI 10)—that public institutions have had very limited capacity to monitor compliance with the law. This is due to lack of personnel for effective company oversight, insufficient technical knowledge to analyze and supervise algorithm functioning, challenges involved in supervising an atypical and previously non-existent figure such as an independent worker without an employment contract but with protections defined in the labor code, the territorially dispersed nature of the work process, among others. As a union leader of platform drivers commented: “*You go to the labor inspection, anyone, and the official tells you “you have no rights,” or “you know what… I have no idea what to do, I don't know how to act.” That is the response from the labor inspection official 2 years after the law's publication*” (KI 2). To date, two oversight programs have been carried out by the Labor Directorate, focused on delivery companies, which have centered on health and safety issues, verification of service contracts, proportional minimum wage payment, transparency in data access and algorithm functioning [Dirección del Trabajo (DT), 2025; Ministry of Labor and Social Welfare (MTPS), 2025]. However, after almost 3 years of law implementation, no specific oversight plans have been developed with a focus on determining whether—for some groups of workers—the signs of employment relationship established in Labor Directorate ruling 1831/39 are met or not, which if confirmed would require companies to change the contracts (from independent to dependent) of those workers. One of the key state informants interviewed (KI 9) indicated that oversight of this aspect of the law faces the risk of massive judicialization of cases by companies, as well as the possibility that under a new upcoming government and a new Labor Directorate, the aforementioned ruling that currently interprets this aspect of the law could be reviewed and modified in a more lax sense regarding employment relationship indicators. In this context, the State's focus has been on advancing formalization and access to social protection for independent workers—in which there have been important advances as mentioned—and on addressing health and safety issues, and more recently matters related to transparency of criteria and providing clear information to workers about how rates and payments are determined.

Fourth, and regarding the more concrete impact of the law on working conditions, evaluation studies conducted 1 or 2 years after the law entered into force point to limited scope and ambivalent results: on the one hand, there is evidence—especially among drivers—of low knowledge of the law and its implications, persistent issues regarding the lack of clarity in payment criteria and workers' ability to verify that these are correct, insufficient or non-existent communication channels (physical offices in each city with company staff providing service), arbitrary company decisions (account suspension or blocking), lack of clarity and difficulty accessing the insurance defined by the law, among others (FLACSO, 2023; Superior Labor Council, 2024; KI 2, KI 11; Centro de Políticas Públicas UC (CPP-UC), 2023). Some workers also point to decreased income due to tax payments deducted by companies as a negative impact, which has not been balanced by improved rates and—in the case of drivers—a reduction in the commission charged by platforms. On the other hand, among its positive impacts are -without doubt- the greater protection in occupational health and safety, both due to the express responsibility of companies in this area (Article 152 quinquies F, Law No. 21,431), as well as the progressive formalization of workers through the issuance of fee invoices and payment of mandatory contributions from companies (Gontero and Ravest, 2024; Superior Labor Council, 2024). As a worker tell us regarding the formalization process and access to social protection:

I mean, it's positive in the sense that you're going to have, you're going to be able to access FONASA [Public health system], you won't have a gap in your AFP [Pension fund] because that money is going to go there, but the negative thing is that you have to cover that cost yourself, because Uber deducts it entirely from you, from the trip fare. It's not like Uber as an employer in this case takes it on themselves, or that Uber charges it to the customer, no, you as a driver assume it directly ” (Male Driver, Chilean, 37 years old, Concepción).

#### 3.2.2 Ley 21.553: regulation of ride-hailing platforms

The labor market for delivery and ride-sourcing platforms in Chile is regulated not only by the Labor Code (Law 21.431), legislation related to social protection for independent workers (Law 21.133), and rulings from the Internal Revenue Service (Resolution 132)—as discussed in the previous section—but also by specific sectoral legislation, such as Law 21.553, which was approved in Chile in April 2023, although its implementing regulations were only issued in April 2025. The focus of this law—commonly known as the Uber Law, and set to come into effect in 2025—is the regulation of transportation app companies such as Uber, Cabify, Didi, and others. The law and its implementing regulations establish a set of requirements, of which the following are particularly noteworthy. Companies are required to: be incorporated as legal entities in Chile; operate only with drivers registered and authorized in a regional registry maintained by the Ministry of Transportation; contract insurance covering vehicles, drivers, passengers, and third parties; provide detailed information to passengers using the service; supply drivers with information about the passenger's name and rating, proposed route, service time and fare, and payment method; and offer clear information about the criteria for registration, blocking, and suspension of driver accounts, as well as the criteria for evaluating and ranking both passengers and drivers.

Regarding drivers, the law requires registration in the driver registry, possession of a professional license, and a clean criminal and traffic record. For vehicles, requirements include passing technical inspections every 6 months, having four doors, automatic locks, a minimum engine power of 1.4, a maximum age of 7 years at the time of registration, valid insurance, and displaying a QR code sticker on the windshield that allows quick verification of all relevant documentation, among other conditions.

At the origin of this law, whose discussion dates back to 2016, are the complaints from traditional taxi associations against Uber and other app companies, considering them unfair competition given the low fares and the lack of requirement to comply with various usual requirements in the passenger transportation sector (professional license, vehicle age and safety standards, driver registration, etc.). In 2016, episodes of violence by taxi drivers against app cars and drivers were recorded, as well as massive street protests by taxi drivers, in a manner similar to what occurred in other parts of the world. This conflict forcefully brought the discussion about regulating app-based transportation companies onto the public agenda, and it is interesting to note that it was this issue and not the debate about the contractual status of workers that inaugurated in Chile the discussion about regulations for digital work platforms.

Despite the fact that early in 2016 a proposal for regulating passenger transportation platforms was presented by the government of the time (Bill 10.937-15), the law was only approved in 2023 and its regulations in 2025. The law that was finally approved is based on a second bill presented in 2018 by the government of the time (a right-wing coalition) and which—unlike the first proposal—did not include the establishment of minimum and sustainable fares. This had been a central demand of app drivers, and was also part of the agreement signed between them and the taxi unions within the framework of discussions on how to regulate the sector.

Throughout the entire process of discussing the law, and subsequently the regulations, companies like Uber carried out strong media campaigns against such regulations, stating that these would produce a rise in fares, a decrease in service availability for users, and job losses for many drivers who would not be able to comply with the new law requirements. Uber has stated that especially the requirement for maximum vehicle age “will directly affect the supply of cars available for people, quality of service and therefore the cost of trips” (La Tercera, 2025), in addition -together with other companies like Cabify- to wielding the argument of the safeguarding of information and privacy of passenger and driver data that the new law would affect.

It is interesting to note that since its inception in 2012, the work of app drivers has not been considered a legal activity, with drivers being subject to fines from inspectors and police, and sometimes having their vehicles impounded for not complying with the current passenger transportation law. To this day, Uber drivers, given the delay in implementing the “Uber law” regulation, are exposed to fines and vehicle recovery charges that can reach USD 800, despite the massive use of such applications in society and the regular payment of taxes they make to the State as a result of the regulation of law 21.431 discussed above. This demonstrates the multiplicity of regulations—labor laws, economic sector regulations, tax laws, and even immigration laws—that have gradually shaped Chile's digital platform labor market. It also reveals their lack of coherence among themselves, and how the approval processes of these various laws reflect the interests and bargaining power of different actors, political contexts, and the contingent nature of social processes.

Returning to Law 21.553 on the regulation of ride-hailing platforms and its upcoming implementation in 2025, the law will significantly impact this labor market. This will especially affect drivers who must exit the market due to lacking professional licenses or vehicles that meet the new requirements, representing a fundamental change to the traditionally low barriers to entry in this labor market. A group of the interviewed workers express their distrust and rejection of the law, or some of its aspects -especially the professional license requirement, the regionalization of the driver registry, some of the vehicle requirements-, and they fear how this might affect the flexibility and autonomy with which they currently operate on the platforms, or lead to a decrease in customer demand due to an increase in fares, or directly force them to leave the sector. However, an important group of the interviewed workers -especially those who have been dedicated to this work as their main activity for several years- indicates their support for the regulation insofar as it will allow them to work without the risk of fines and vehicle impoundment, will provide more security by having driver and user registries, could produce (by having fewer vehicles) a rise in fares that improves drivers' income, and would contribute to professionalization and improvement of service quality based on professional license requirements and the use of safer, more comfortable and modern vehicles. As an interviewed driver tells us.

I feel that regulation is always good… for example, the issue of these same cars that are small (…) passengers don't like getting into small cars, and they always tell me that: “how good that your car is big.”… (…) The other thing is that fares are going to go up. That's something that's expected. When this starts, fares are going to go up, because there won't be so many vehicles anymore. The smaller vehicles are going to go out of circulation. And only the drivers who meet the requirements are going to remain. (…) The other thing is that one is going to have to get a professional license. So, also, it's a whole issue of one being more qualified…to do this job…. I feel that it's going to give me the right to be able to work with more security and with whoever I want. (Male driver, Chilean, 30 years old, Santiago)

### 3.3 Workers: sociability and collective organization and action

Understanding digital delivery and ride-hailing platform labor markets requires—alongside the role of companies and the State—analyzing the sector's workers, not only in terms of their socio-demographic profiles, but by considering their meaning-making constructions, identity narratives, logics of action, and forms of collective organization. These different aspects are not presented homogeneously, given the noted differences between delivery and passenger transportation platforms, and considering also the diversity of subjects who participate in this labor market (according to age, social class, nationality, gender, care responsibilities, educational level, etc.), who in turn connect to platforms under very dissimilar circumstances (professional job loss, arrival as an undocumented immigrant, activity parallel to studies, etc.) and with variable time commitments (Reygadas, 2020).

Within the framework of this article—and having addressed the topics of meaning-making constructions and identity narratives in previous publications (Morales and Stecher, 2023; Stecher and Morales, 2024)—we focus on the axes of sociability and organization and collective action, of particular importance for understanding the current configuration of these labor markets in Chile. Without ignoring existing heterogeneity, we account for certain patterns that are transversally shared and relatively stabilized among workers on both platforms analyzed.

#### 3.3.1 Sociability and everyday bonds among platform workers

One of the defining characteristics of work mediated by digital platforms is its fragmentation and individualization. In the absence of work centers and an employment relationship that favors recognition as peers, and given the wide geographical dispersion that companies organize through the individual interaction of the worker with the app, the platform model atomizes and isolates workers, often making them compete with each other and hindering the possibilities of associativity and collective organization (Graham, 2020; Vandaele, 2018).

However, in the case of passenger transport and delivery platforms, national and international studies have repeatedly shown that, despite the fragmentation and individualization of work, the geolocalized nature of the service provided implies the encounter of workers in the performance of work, which allows processes of recognition as peers and the development of forms of mutual support that have different meanings and orientations. Our research confirms these findings, which are illustrated in the following testimonies:

Look, when I can arrive at a restaurant that's in Ñuñoa, and it turns out there are several, there are 10 delivery workers waiting for orders, and among those there are girls and boys and I don't know them, but we always greet each other, as delivery workers we know we are colleagues, I mean it's like we are like a brotherhood, if we see each other on the street affected by something, what happens? can we help you? if we have tools, I mean among ourselves we support each other and the majority, the majority of delivery workers in this country are Venezuelan.” (Woman delivery, 30 years old, Venezuelan)

I said, “I can't be the only one doing this! So when I was waiting I would look at the cars to see who was looking at the cell phone, the app… If I saw someone I would say “hey, friend, are you an Uber driver? And then we would start talking (Male driver).

As our interviewees report, workers actively seek out others with whom they can share experiences, resolve doubts, and—when contact networks and more stable groups form— protect themselves from specific risks and feel accompanied in their work.

For delivery drivers, using backpacks and jackets with company advertising allows them to be recognized as peers. The predetermined connection areas for receiving orders, the rest areas set up by some companies in certain parts of the city, or the outskirts of the stores and restaurants with the highest demand become meeting places where, while waiting, they begin to get to know each other and exchange their work experiences. In the case of the delivery workers, these exchanges often involve sharing experiences of immigration, arrival and insertion in the host country.

These first exchanges often lead to the formation of WhatsApp groups (or other messaging applications) among delivery workers and drivers. Through these groups, they extend the face-to-face meeting space to the virtual one, allowing them to weave a support network to share tips on how to use the platforms and maximize income, help each other solve unforeseen events or problems during the job, share their experiences, or simply keep each other company throughout the workday.

These forms of sociability can be understood as embryonic solidarity (Atzeni, 2014) derived from the work process itself: to provide the services properly each worker must learn to use the apps, to solve the unforeseen events that arise and to make decisions that allow them to complete the services in the shortest possible time and maximize their productivity. This is not necessarily contradictory to the companies' objectives, as they benefit from this induction process that peers do in the tricks of the trade to new workers, in a context in which companies do not mobilize resources for formal induction and training processes. In this sense, there is a sociability marked by a form of solidarity that provides support to workers, while at the same time, it is not antagonistic to the company, but favorable to it. An example of this is found in the self-organization of Uber drivers to respond to service demands:

“We for example in the group (of whatsapp) that I am we are 50 drivers, to be able to provide help in case of any emergency and we also give each other news of how many cars of each group can be when there is much demand (…) we are organizing ourselves to be in the strategic points where there are more requests in the application” (Male driver).

In addition, as the above quote shows, these groups address needs that companies do not meet, particularly with regard to safety. Thus, helping each other in the event of assaults or accidents, recovering stolen vehicles in the face of police inaction, or advising each other on insurance or other prevention and protection measures are common practices that are discussed and coordinated through these channels. Indeed, after the increase in assaults on delivery workers due to the pandemic and the perceived defenselessness, groups of delivery workers organized themselves for the “recovery” of stolen motorcycles, which ended in several episodes of street violence and was the subject of debate in the media (Chilevisión, 2020). As an interviewee pointed out, these groups play a key role as fundamental support and protection mechanisms on a daily basis:

…my coworkers are more of a brotherhood, we all try to take care of each other, if something happens to any of us, we can go help each other and we go and meet with him to help each other, we always try to go up as a group and come down as a group, we always get in touch with our coworkers (Male delivery worker, immigrant, 34 years old).

On the other hand, along with these “closed” face-to-face and virtual spaces limited to specific groups of workers who meet and coordinate at certain times and sectors, there are also pages on social networks where workers and people interested in using the apps raise issues of all kinds: from renting accounts or vehicles to drive or deliver, to complaints about the usual problems. Facebook or Instagram pages such as “Soy Rappi Chile,” “Ni un repartidor menos Chile,” or “Conductores de Apps Chile” have been some of the most used and have several thousand followers, although in recent years they have decreased their activity.

Some of these support groups or virtual networks are typical of immigrant delivery workers, among whom there is a strong group identification and solidarity aimed at helping each other improve their working and living conditions within the framework of their migratory trajectories.

Thus, as other studies have shown (Gutiérrez and Atzeni, 2022), in Chile, there is solidarity among platform workers, which is highly valued by them, but this is not necessarily antagonistic to the companies, nor does it necessarily imply a greater willingness to make demands on companies regarding working conditions.

#### 3.3.2 Collective organization and action

Alongside these support networks and informal spaces of sociability—both face-to-face and online—it is also possible to identify in Chile the presence of organizations and forms of collective action by platform workers that have raised claims and protests directly against the companies, and which had significant weight and visibility—as was previously noted—in the context of the pandemic. Various protests concerning precarious working conditions and legal actions brought before the courts constituted during this period the context of heightened activism and protest in Chile. Several associations were established between 2018 and 2020—Ni un repartidor menos, Agrupación de repartidores penquistas, Marea—which participated in various protest and denunciation actions in the courts, including participation in international calls for work stoppages (Gutiérrez and Atzeni, 2022).

These forms of organization and collective action, characterized by varying levels of organization and formality, which have deployed diverse strategies to defend their interests as platform workers, can be understood as an expression of active solidarity (Atzeni, 2014). Some of the central issues of concern and key demands of these groups have included the unilateral and downward modification of fares implemented by companies, account blocking and closure, conditions of insecurity and risk on the streets due to robberies and accidents, worker accountability, and the lack of support for various contingencies that arise with passengers and orders in day-to-day operations. More recently, these concerns have been supplemented by issues related to the implementation of Law 21.431, as well as demands regarding Law 21.553 regulations for ride-hailing drivers. In these cases, demands have been directed toward both companies and the State, given the State's responsibility for oversight (Law 21.431) and for approving and implementing Law 21.553 regulations.

While there was a certain decline in protest actions and legal complaints following the pandemic and the initial implementation of Law 21.431 (2022), along with the disappearance of some organizations, the last 2 years (2024-2025) have witnessed a certain re-articulation and increased activity among worker organizations. Driver unions −11 at the national level with approximately 12,000 members—affiliated with the Federation of Application Drivers of Chile (Federación GRECO App Chile), Union No. 1 of Independent Workers of Application Drivers (SINCAPP), the more recent Platform Workers Union (SINTAPP), as well as the Secretariat of New Forms of Work of the Unified Workers' Central of Chile (CUT) have achieved greater prominence over the past 2 years—vis-à-vis workers themselves, companies, and the State—within the framework of legislative discussions in parliament and social dialogue forums promoted by the Ministry of Labor, both associated with the processes of implementation and evaluation of the new regulations.

Thus, for example, on April 29, 2024, some of these organizations called for national mobilizations—an app blackout—with a focus on improving driver fares and earnings. As one ride-hailing drivers association leader explained (KI 2):

Look, even for April 29, apart from holding a strike, we held a mobilization in some regions. It was done in Puerto Montt, it was done in Concepción, well, in Concepción we have always done it, but that is another matter. It was done here in Valparaíso, in Chillán, and in Talca (…) The general strike, as I say, 60% [of adhesion]… (…) And believe me, we are fighting for a possible second strike, because things look and continue to be bad economically for us. (…) Our operating cost, depending on the vehicle, our cost per kilometer is between $350 and $390…and the applications are paying between $280 and $300. Then, as people have been realizing, and this has also been our fault in a way, but in a good way, that making people see that they are losing money in each trip is the great discontent that exists today.

Our data captures these organizational processes since 2019, which have been dynamic and with some differences between drivers and delivery workers. The Table 2 shows the formal and informal collectives of platform drivers and delivery workers oriented to demand and defend their interests vis-à-vis companies or vis-à-vis the State.

**Table 2:** Formal and informal collectives of platform drivers and delivery workers.

**Dimensions**	**Drivers**	**Delivery workers**
Informal collectives through messaging groups or other virtual and face-to-face channels	Yes Local groups that in some cases become formalized as trade associations	Yes Rappi 08 Riders United Now Not one delivery driver less Chile Mancomunal de Repartidores de Aplicación (MAREA)
Formal organizations	Yes ACUA, GRECCO, AGECOP, COTRAP (Trade associations) Uber RV Union Independent Union of APP Drivers (SINCAPP) Platform Workers Union (SINTAPP)	Yes Agrupación de Repartidores Penquistas (community association) Cornershop-Uber Union (Initially created as a delivery workers' union, currently, after the purchase of Cornershop by Uber, it is a union of administrative workers with an employment contract from the company) Platform Workers Union (SINTAPP)
Public protests	No	Yes March 2019 call in Santiago for Uber Eats Delivery Drivers April 2020 International Strike for app delivery drivers
Mass disconnections as a form of protest	Yes	Yes
International alliances	Yes International Alliance of App-Based Transport Workers (IAATW)	Yes United World Action
Main focus of its collective action	Impact on the legislative debate Improved rates Security against theft	Negotiation with companies for labor improvements Demands to the State for oversight of Law 21,431, especially with regard to workplace safety, unilateral disconnection, blocking of accounts, and clarification of payment calculation criteria

Analyzing in more detail and separately these organizations of delivery workers and app drivers it is possible to point out the following.

In this 2019–2025 period, the active role of some associations of ride-hailing drivers in the legislative debates on the laws and regulations of the sector stands out. After the null response they had from companies such as Uber regarding specific situations that affected them negatively (high commissions, deactivation of accounts, lower fares), they decided to focus on influencing the regulatory debate on transportation platforms that was taking place since 2016 in the National Congress. To this end, they formalized themselves as trade associations—a figure intended mainly for companies (Ministry of Labour and Social Welfare, 1979)—participated in various instances of pre-legislative debate, met with parliamentarians and government authorities, and even allied with the main cab drivers' associations to propose that the law establish a minimum fare for services to end Uber's dumping on the sector. The latter is a particularity of the Chilean case in view of the confrontation that marks the relationship between cab drivers and platform drivers at international level.

[The agreement with the cab drivers] cost, I'm not going to say it didn't cost, but it was super nice. Before you could not even think about this because there were other leaders who said let's go to the fight. But there was a death! There was a death and the authorities did not do anything (Leader of App Drivers'Association).

Internationally, they established networks through instant messaging channels with organizations of platform drivers from various countries, and in 2021, they participated through ACUA (one of the most visible trade associations) in forming the International Alliance of App-based Transport Workers. It should be noted that drivers do not stand out for the use of massive forms of protest in the streets, although they do carry out “strikes” in the form of temporary disconnection from the platforms. The first of these initiatives occurred in May 2019, when they adhered to the Global Strike called by U.S. collectives on the occasion of Uber's IPO, and subsequently, in 2023 and 2024 the SINCAPP Union has again called this type of strike to press for an increase in service fees. Due to the quality of this type of action, there is no clear record of the level of adhesion.

Between 2023 and 2025, the GRECO Federation and its affiliated unions—established from September 2022 under the collective rights granted by Law 21.431 to independent platform workers—have played a highly active role in parliamentary discussions regarding the pending regulations for implementing Law 21.553. They have expressed concern about delays in the approval and implementation of these regulations, noting how such delays maintain them in a state of illegality and expose them to fines and penalties of up to $800, while also perpetuating risk levels for robberies and even murders, given that the driver and passenger registration and identification system established by the law has not been activated (IC 2). They have also continuously informed their members about the legislative debate and regulatory approval process through various social media videos.

On the other hand, the collective action of delivery workers has stood out especially for including forms of mass protest in the streets in the face of worsening working conditions (Unilateral change in criteria and reduction of rates, risk of accidents and thefts, lack of basic services such as bathrooms or places to eat, risk of contagion in the context of the pandemic, etc.). The first of these demonstrations was led by Uber Eats delivery drivers in 2019, who went to the company's offices without being received, and the second in 2020 by Pedidos Ya delivery drivers, with a similar outcome. In both cases, these protests were self-organized through instant messaging groups by informal collectives.

In February (of 2020) we began a communication campaign that we carried out through WhatsApp groups, from anonymity (…) in April we received an email from the company saying that they were going to restructure the payments and that they were again reducing the salary, they totally reduced it, which translates into more working hours, more workload but less money (…). At that point and in response to a call from an international strike of delivery drivers from Argentina, the ATR movement and Brazil, from Latin America, we decided to call a mobilization where we went public and we gave ourselves the name of Riders United Now and we called two mobilizations 2 days in a row, with total success, we went to the television stations and they covered our campaign, more than 600 motorcyclists (…). From there we stayed in the streets and started this struggle that we will continue to carry on because we want to recognize benefits for the workers and that labor rights are recognized (…) (union delivery workers' leader).

These protests ended with the arbitrary deactivation of the accounts of some of the most visible delivery drivers. With the support of the main Central de Trabajadores CUT, they filed two class action lawsuits against PedidosYa for anti-union practices and protection of their rights as employees, and together with other delivery drivers' collectives, in 2020 they formed an informal federation called Mancomunal de Repartidores de Aplicación, MAREA. At the same time, another collective in Concepción, Agrupación de Repartidores Unidos, was formalized as a community group (a figure intended for organizations of specific interest in a city) and, after being blocked by Pedidos Ya, they also sued the company and tried to form a cooperative of delivery drivers, following the example of Coopcycle in Europe. They all continued to participate in the international network Unidxs World Action through virtual assemblies and digital propaganda actions.

With the end of the pandemic and the implementation of Law 21.431 (September 2022), which legitimized the status of independent worker, both street protests and court cases filed by delivery workers declined sharply, and many of the aforementioned organizations disappeared or weakened. However, in the last 2 years, under the auspices of the Ministry of Labor's interest in promoting social dialogue initiatives, and with support from the CUT, some new delivery worker unions have been created in different cities within the framework established by the same law. These are still very weak organizations whose focus has been on accompanying the law's implementation process and continuing to demand improvements in basic working conditions. Some of these demands have been presented directly to companies through communication channels opened by these social dialogue initiatives.

It is important to emphasize that beyond this relative resurgence of collective action and organization among platform workers over the past 2 years, it remains extremely weak and fragile. Structural, associational, and social power resources are scarce, with their action currently leveraged on a certain margin of institutional power derived from the processes of discussion and implementation of the aforementioned laws (Schmalz, 2017). As two interviewed leaders told us, these are unions with low membership rates, no payment of dues—although these have been established in the statutes—no regular assemblies, high member turnover, considerable fear among workers—especially those with irregular immigration status—of losing their sole source of income, and a strongly instrumental orientation among workers toward improving income and benefits, which is exploited by companies when it comes to organizing protests or mass disconnections. As one interviewed union leader told us (KI 11):

No, it hasn't been done [an assembly]. One day I wanted to call one online and we ended up with four people connecting, and I don't… it's just very difficult… I mean, as a union as such, as you are accustomed to, nothing like that. This is totally atypical and it's been very difficult, it's very difficult… first, time [lack of time]; second, (…) there's a horrible fear, I tell my mom, it's incredible how these guys are so afraid that they'll block your account, that they'll close your account, that for any reason or if the company finds out that you're part of the union they'll close my account… here everyone protests, but it's purely for money, for fares, fares, fares, fares… So I tried to organize a mobilization [in 2025], but look, for example, in my area the longest we stayed on strike was 2 h, and that was extremely difficult. Because the app is alive, the app knows there are people connected but not accepting orders, so what does the app do? It throws you an order at a higher amount, and so these guys were anxious thinking “damn, they threw me an order for 15, for 20 and it keeps going up…” and they were desperate.

It should be noted, finally, that for both driver and delivery worker organizations, the issue of laboralization and the demand for an employment contract as a dependent worker does not appear on the horizon of their immediate demands. In the case of drivers, there is even—something that has not changed since the arrival of platforms—an active and explicit rejection of the possibility that this legal framework would be applied to them.

## 4 Conclusion

The article has presented a reconstruction of the digital delivery and ride-hailing platform labor market in Chile based on three axes of analysis: (i) companies and their business models; (ii) State laws and institutional regulations; and (iii) workers and their dynamics of sociability, associativity and collective organization. The reconstruction allows us to account for the historicity, dynamism, transformation, and multiplicity of actors that constitute the digital platforms labor market in Chilean society today, highlighting how these dimensions overlap and mutually condition each other.

This reconstruction enables an understanding of how this labor market reproduces global patterns of the platform economy model. Simultaneously, these patterns exhibit specific variations due to the historical fragility of formal labor markets in Latin America and the extensive nature of their informal sectors. They are further shaped by particular Chilean circumstances, including massive Venezuelan immigration, the profound institutional and cultural impacts of decades-long neoliberalization processes, and recent regulatory initiatives partly linked to the socio-political context created by the social unrest and the pandemic.

Likewise, the reconstruction offered for the Chilean case confirms, illustrates, and enriches some of the central theses of recent debate in Latin America on digital labor platforms. Our findings reveal, first, a consolidated and expanding labor market; second, one where companies' business models are based on and expand previous logics of neoliberalization, flexibilization, and precarization of work and employment; and third, a labor market that acquires distinctive characteristics specific to Latin America as a result of the historical fragility of formal labor markets as social inclusion mechanisms, the extension of the informal sector, and the privatization and individualization of social protections that occurred under the neoliberal modernization model.

In terms of a broader conclusive analysis, it is possible to propose that this labor market can be characterized by three principal features: *precariousness, heterogeneity*, and the capacity to facilitate *fragile forms of social inclusion*.

On the one hand, despite very recent advances in formalization and social protection introduced by recent legislation, these constitute precarious labor markets characterized by the absence of employment contracts and viable collective bargaining mechanisms with companies regarding working conditions. This precarity manifests through companies' unilateral authority to define and modify compensation rates, creating an environment of uncertainty and pronounced information asymmetries. Additional factors include the lack of formal compensation for idle time or waiting periods between task assignments, significant occupational health and safety risks stemming from extended working hours, night shifts, accident risks (particularly for motorcycle delivery workers), assaults and robberies, inadequate restroom facilities, and harassment, violence, and mistreatment by customers or restaurants. Further risks include potential fines—until the implementation of the so-called Uber law—imposed on ride-hailing drivers for operating what is considered non-regulated transport services, and penalties affecting delivery workers in irregular immigration status who lack national driver's licenses.

It is a labor market that feeds on the structural fragility of the formal labor market and the naturalization of the informal economy sector, nourishing itself on—and reproducing—structural conditions of labor precariousness throughout society as a whole. Law No. 21.432, despite the important advances it represents in the social protection of workers, does not structurally alter this dynamic. On the contrary, the institutionalization of a labor category excluded from labor law protection, as well as the inability of public institutions and the fragility of unions to monitor its proper implementation, may serve as a precedent for the lack of protection of new worker segments both within and beyond the platform economy.

Secondly, this labor market, while sharing structural patterns in its productive and business models across companies operating both in Chile and globally, exhibits considerable internal heterogeneity. The data reveal variability between delivery and rideshare companies across different aspects such as workforce composition—particularly regarding the proportion of immigrant workers; work processes, influenced by the presence of restaurants and retail establishments in the case of delivery workers; and levels of control and task monitoring, which are more pronounced in delivery services. Additionally, workers in both sectors display distinct cultural orientations, with drivers demonstrating greater identification with independent and entrepreneurial work profiles. The sectors also differ in their collective organization strategies, with drivers emphasizing legislative advocacy while delivery workers favor direct street action. This heterogeneity extends to platforms within the same category, manifesting in divergent worker onboarding requirements, identity verification mechanisms, payment structures, and -for a limited period- labor relations models—as exemplified by early Cornershop's distinctive approach. Furthermore, cross-cutting segmentations exist throughout the entire labor market, transcending platform types and companies. These segmentations are characterized by diverse worker profiles and heterogeneous groups who enter platform work due to varying structural constraints and subjective motivations, including immigrants, unemployed professionals, and women with caregiving responsibilities, among others.

Finally, it is a labor market that offers possibilities of precarious or tenuous social inclusion to segments of the working population that risk deeper and more severe conditions of exclusion. These possibilities have been enhanced by recently enacted legislation. Despite the precarious conditions outlined above and corporate exploitation of these arrangements to reduce labor costs while maximizing profits, platform economies nonetheless provide valued spaces for workers seeking labor market insertion, social inclusion, income generation, and certain degrees of autonomy and flexibility. This valuation is particularly pronounced within a context characterized by poor-quality formal employment and the extensive privatization and commodification of social rights, alongside neoliberal cultural imaginaries that position individuals as solely responsible for managing their own conditions of social reproduction. This dynamic of fragile social inclusion which, while feeding and reproducing labor and institutional precariousness in the countries of the global South, offers a temporary buffer or lifeline to it, is one of the characteristic features of this labor market.

Labor precariousness, heterogeneity, and fragile social inclusion thus constitute structuring and structural features of the labor market of delivery and transport platform companies in Chile, and pose specific challenges for workers' organizations and for the construction of public policies and regulations that allow for better and fairer working conditions in this sector. As our findings demonstrate, this represents a dynamic labor market characterized by processes of transformation and stabilization over time, shaped by global structural and systemic forces that condition its operation, while simultaneously being subject to local socio-political contexts and the agency of diverse entrepreneurial, state, and worker collective actors.

## Data Availability

The datasets presented in this article are not readily available because data are confidential and anonymous. Requests to access the datasets should be directed to antonio.stecher@udp.cl.
